# Adult ADHD Symptoms in Syrian War Refugees with Long-Term Health Conditions: A Screening Cross-Sectional Analysis from Jordan

**DOI:** 10.3390/healthcare14091174

**Published:** 2026-04-28

**Authors:** Omar Gammoh, Alaa A. A. Aljabali, Sireen Abdul Rahim Shilbayeh, Mariam Al-Ameri

**Affiliations:** 1 Department of Clinical Pharmacy and Pharmacy Practice, Faculty of Pharmacy, Yarmouk University, Irbid 21163, Jordan; 2 Department of Pharmaceutics and Pharmaceutical Technology, Faculty of Pharmacy, Yarmouk University, Irbid 21163, Jordan; 3 Department of Pharmacy Practice, College of Pharmacy, Princess Nourah bint Abdulrahman University, P.O. Box 84428, Riyadh 11671, Saudi Arabia

**Keywords:** adult ADHD, refugee populations, diabetes

## Abstract

Background/Objectives: Attention-deficit hyperactivity disorder (ADHD) in adults is often overlooked in refugees, especially those displaced by war and diagnosed with chronic issues such as hypertension and type 2 diabetes mellitus. We sought to provide a preliminary screening to align with ADHD screening and to examine its related demographic and clinical factors. Methods: This cross-sectional study recruited Syrian refugees residing in Jordan. The Adult ADHD Self-Report Scale-V1.1 (ASRS) was employed to provide a preliminary screening of “Consistency with ADHD screening”. Multivariable regression analysis was used to identify the risk factors associated with the “Consistency with ADHD screening”. Results: Data analysis included 345 patients; 179 (51.9%) were females. β-Blockers were used in 151 (43.8%), metformin in 134 (38.8%), and sulfonylurea in 86 (24.9%). In the study, 158 participants (45.8%) reported very consistent results related to ADHD. We used multivariate binary logistic regression, which showed that in our groups that received metformin (OR of 2.08, 95% CI 1.32–3.27, *p* = 0.001) and insulin (OR of 2.25, 95% CI 1.00–5.04, *p* = 0.04), we observed a positive association with ADHD symptoms. Also, we noted that high school education was negatively associated with the results of the ADHD screen (OR of 0.58, 95% CI of 0.36–0.94, *p* = 0.02). Conclusions: This preliminary study guides future steps in addressing ADHD symptoms in war-displaced refugees; proper diabetes management and education seem to be important factors.

## 1. Introduction

Attention Deficit Hyperactivity Disorder (ADHD) is common in children which is what it is often seen as, but what we are seeing via research is its great presence and persistence into adult years. In adults, we see different presentations of the disorder which includes elements of impulsivity, disorganization, difficulty with attention and impact in daily function [1]. Also to note is that refugees which are alreadymentally vulnerable because of the issues related to war and displacement may be at higher risk for a range of mental health issues which may include ADHD [2].

Refugees going through crises, such as wartime displacement, present with a variety of stressors, which in turn may bring out or worsen attention issues. The disruption of routine life, exposure to trauma, and uncertainty about the future may heighten attention and impulse control problems [3]. In refugee settings, we also see resource scarcity, which may reduce access to proper diagnosis and treatment, further complicating the situation. Therefore, it is of great importance to research and implement solutions for Adult ADHD in refugee groups, which in turn will improve their overall well-being and how they adapt to their new environment [2]. We must also examine the connection between ADHD and war-induced displacement and adult health issues, which is very important for accurate healthcare planning, complete mental health support, policy formation, research, and better public health responses during crises. In the case of adult ADHD, unlike that of childhood, which is diagnosed early, in adults, it often goes undiagnosed, which in turn affects how they function in many areas of life, particularly in refugee and social out-groups, where the issues of displacement are a big issue. Also, the fact that there is a presence of undiagnosed or untreated adult ADHD greatly hinders adaptation and mental well-being [4]. In our study, we also noted a connection between ADHD and heart disease, which in turn brings out the need for a comprehensive healthcare approach for refugee patients. This will require us to tailor our medical care to the specific needs of each individual, as the relationship between ADHD and cardiovascular diseases is very much so. What we see is that the relationship between ADHD and cardiovascular disease is also very complex.

Hypertension and type-2 diabetes are among the most commonly reported diseases among refugees worldwide, and those residing in Jordan [5]. It is noted that adults with ADHD present a large at-risk group for these diseases, which in part is a result of lifestyle factors related to the disorder, which in turn lead to poor health choices. Also, in the case of refugees who have a high comorbidity of ADHD and cardiovascular disease, this presents a healthcare challenge, which in turn calls for a very integrated healthcare approach [6]. Also, in the case of refugees who have a high tendency of also having ADHD and cardiovascular disease, we see this as a healthcare issue, which in turn calls for a very integrated healthcare approach.

The coexistence of ADHD symptoms and type 2 diabetes seems an important merging topic. One cross-sectional study recruiting 315 patients reported that about one-half of people with type 2 diabetes met the symptoms of ADHD diagnostic criteria using the Adult Self-Report Scale V1.1 (ASRS), furthermore, 3.6% of the participants were already diagnosed with adult ADHD [7]. In addition, another large-scale investigation involving >5 million adults revealed that adults diagnosed with ADHD reported a higher prevalence of type 2 diabetes versus those without ADHD (3.9%) versus (1.6%), respectively. The same study confirmed that adults with ADHD reported a higher prevalence of hypertension than those without ADHD [8]. One possible explanation of this association is unhealthy lifestyles, such as poor eating habits and a lack of physical exercise [9], which could represent a common background between type-2 diabetes and ADHD symptoms.

ADHD drugs are reported to have a very close tie with cardiovascular issues, which, at the same time, is a matter of debate [10,11]. In addition, the issue of how ADHD, cardiovascular disease, and the unique issues faced by refugees during crises, such as war displacement, come together to form a complex whole that requires study.

In this study, we present a first look at screening for adult ADHD symptoms in this crisis, which has caused displacement of our population, which also has hypertension and type 2 diabetes, and at the same time, we are looking at the issue of what the medications we are giving them may be affecting. It has been suggested that demography and drug use may play a role in screening individuals for ADHD.

To our knowledge, very little research has been conducted to determine the prevalence and identify risk factors for ADHD symptoms in Syrian refugees, including those who have been diagnosed with cardiovascular and related diseases, such as hypertension and diabetes. It can be hypothesized that ADHD screening could be related to disease severity or medication use in addition to other demographics.

Thus, our study aimed to do a primary screen for ADHD symptoms and at the same time to look at what demographic and clinical factors play a role in the report of these symptoms in a group of Syrian refugees who also have a diagnosis of hypertension and diabetes.

## 2. Materials and Methods

### 2.1. Study Design and Patient Recruitment

This study employed a cross-sectional design and used predefined inclusion criteria. This investigation is a part of a big research project focusing on mental health that was approved by Yarmouk University Institutional Review Board (IRB), no. (692). Syrian refugees attending Caritas (a Catholic non-governmental organization) primary care centers were approached based on the available database for patients diagnosed with hypertension and type 2 diabetes. Data were collected between January and February 2024. The study questionnaire was uploaded to a Google Form. Potential participants were asked to enroll after the study objective and methodology were explained over the phone. All participants had the right to refuse to complete the questionnaire at any time. Also, all the participants signed an electronic consent form before their enrollment. The database comprised 1100 registered patients; 575 were disconnected, 115 declined to participate, and 65 did not meet the inclusion criteria or did not complete the questionnaire; therefore, data were analyzed from 345 patients, yielding a participation rate of 31%. Our previous studies informed the sample size calculation [12,13].

### 2.2. Inclusion Criteria

Syrian refugees who have lived in Jordan for over ten years, are unemployed, have been diagnosed with type 2 diabetes and hypertension, are receiving long-term treatment, and who fully completed the study instrument were included.

### 2.3. Study Instrument

#### Covariates

The demographic section gathered information on participants’ sex, age, education level, and current residence, specifically whether they lived in Amman, Jordan’s capital, or outside it. The clinical section included questions regarding the medications that patients were taking. To ensure the accuracy of the medication data, the authors provided participants with a checklist featuring pictures of medication packages and their brand names in both Arabic and English, sourced from the outpatient pharmacies of Caritas. The medication checklist included metformin, sulfonylureas, insulin, and antihypertensive medication classes.

### 2.4. Outcome Variable

#### ADHD Symptoms Screening

The eighteen-item self-report Adult ADHD Self-Report Scale (ASRS v1.1) was used as the screening tool in this study. This scale was developed by the World Health Organization (WHO) and aligns with the DSM-IV diagnostic criteria for ADHD, demonstrating high internal consistency (Cronbach’s alpha = 0.88) [14]. The ASRS v1.1 consists of two parts: Part A (the screener) comprises six elements most predictive of ADHD; selecting 4 or more boxes in its shaded areas indicates symptoms highly consistent with adult ADHD. Part B contains twelve items for further assessment of ADHD symptoms. The scale has also been previously used in Arabic-speaking populations [15,16].

### 2.5. Data Analysis

Demographics and clinical variables are presented across ADHD screening categories, with frequencies and percentages. A chi-square analysis was conducted to examine the association between the independent variables and the outcome variable, “consistency with ADHD symptoms,” as shown in Table 1. To identify the factors significantly associated with the dependent variable, a multivariable binary logistic regression model was initially built, including the variables presented in Table 1. Using the backward stepwise model, variables with the highest *p*-values were removed first; subsequently, the final model included only independent variables significantly associated with the dependent variable (*p* < 0.05). Data analysis was performed using SPSS software version 23, with significance set at *p* < 0.05 and confidence intervals at 95%.

## 3. Results

### 3.1. Study Sample Features

The study population comprised 345 participants who fully completed the study instrument. Among them, 179 (51.9%) were female, and 186 (53.8%) were over 50 years old. Additionally, 231 participants (66.9%) had completed elementary schooling, and 271 (78.6%) lived outside of Amman. Regarding type-2 diabetes mellitus medication use, metformin was used by 134 (38.8%), sulfonylureas by 86 (24.9%), and insulin by 31 (8.9%). Also, β-blockers were taken by 151 participants (43.8%), ACE inhibitors/angiotensin II receptor blockers (ACEIs/ARBs) by 92 (26.7%), diuretics by 62 (17.9%), and calcium channel blockers (CCBs) by 53 (15.4%). See Table 1 for more details.

### 3.2. Factors Associated with the Consistency of ADHD Symptoms Consistency

The identification of factors associated with the likelihood of ADHD screening was conducted using two levels of analysis. First, a univariate chi-square test was performed to assess the association between each covariate and the outcome variable. All covariates with a *p*-value less than 0.10 were included in the initial multivariable binary logistic regression model. The following four factors were included in this initial model: “age”, “education”, “metformin”, and “insulin”.

The final multivariable binary logistic regression model showed that consistency with ADHD symptoms was positively associated with “receiving metformin” (OR = 2.08, [1.32–3.27], *p* = 0.001) and “receiving insulin” (OR = 2.25, [1.00–5.04], *p* = 0.04). On the other hand, the likelihood for ADHD screening was negatively associated with “high school education” (OR = 0.58, [0.36–0.94], *p* = 0.02). Refer to Table 2 below.

## 4. Discussion

This study aimed to assess the prevalence of consistency with ADHD symptoms and identify its risk factors in a group of Syrian refugees who have been diagnosed with hypertension and diabetes. Our findings indicate that patients receiving metformin and insulin had higher odds of being screened for consistency with ADHD symptoms. Conversely, patients with a high school education had lower odds of being screened for ADHD compared to their peers who received only an elementary education.

ADHD is typically identified in children and can persist into adulthood [17,18]. Based on data from the World Mental Health Surveys, the estimated prevalence of ADHD in adult populations in high-income nations is 3.6% [19]. Meta-analyses show that there has been a significant global increase in the number of ADHD diagnoses [20,21]. Refugees with cardiovascular and related disorders face enormous challenges. However, their problems worsened when they added to the distress of being relocated and not having access to the drugs they needed. This situation affects not only their cardiovascular health but also their mental health, especially if they are predisposed to have ADHD [22]. The relationship between ADHD and heart disease among refugees emphasizes the complicated connection between mental and physical health in at-risk populations [23]. A study conducted on a nationally representative sample of adult Americans concluded that there was a significant correlation between a history of ADHD diagnosis and cardiovascular disease (CVD) [24]. An increased risk of cardiovascular disorders and related disorders has been linked to elevated levels of adult ADHD symptoms [25]. A recent study revealed that people with ADHD have a higher chance of developing a variety of physical health issues, such as cardiovascular diseases [26]. A systematic review and meta-analysis indicated that ADHD is associated with an increased risk of cardiovascular diseases [27]. Findings from the current study revealed that refugees with CVDs who lacked a supply of monthly medications reported higher ADHD severity. Research has indicated that a considerable number of refugees suffer from mental health illnesses that are frequently worsened by traumatic experiences, being uprooted, and having restricted access to medical care [28,29,30]. This situation emphasizes the importance of providing refugee populations with preexisting medical issues and constant access to medication and healthcare services.

We report a significant association between metformin use and consistency with ADHD screening. Metformin, a biguanide insulin sensitizer, is the first-line treatment for type 2 diabetes and is used by almost all patients with type 2 diabetes. According to previous evidence, type-2 diabetes is associated with ADHD symptoms; for example, patients diagnosed with type-2 diabetes are twice as likely to report severe ADHD symptoms and impaired cognition compared to healthy individuals [31,32], which may be due to impaired blood circulation in the frontal and parietal lobes [32]. Although there is no evidence that metformin can affect ADHD or cognition [33]. The authors suggested that this finding could be attributed to the pathophysiology of type 2 diabetes. It can be suggested that ADHD symptoms could be related to the severity of diabetes that required the use of metformin; these findings open horizons for further investigation regarding this issue. According to the literature, ADHD and type 2 diabetes share a common background of a sedentary lifestyle that is very likely to exacerbate both conditions [9].

Similarly, insulin use was associated with consistent ADHD symptoms. The use of insulin in type 2 diabetes reflects the poor glycemic control, which, according to the literature, is associated with ADHD [34]. Therefore, one can not derive conclusions whether insulin or poor glycemic control, or other diabetes-related complications, such as insulin resistance or obesity, or all these factors collectively led to pronounced ADHD symptoms. The co-existence of ADHD symptoms and poor glycemic control could also be attributed to poor concentration, impulsivity, and poor adherence to the treatment regimens and the lifestyle required, as suggested in a recent review [35]. Another suggested explanation is the insulin resistance and the status of low-grade inflammation present in both type-2 diabetes and ADHD symptomatology. Low-grade inflammation could provide a common pathological background to the understanding that uncontrolled glycemic control is more likely to be related to higher ADHD symptoms [36].

In addition, participants who received a high school education as their highest education were at lower odds for ADHD screening compared to their peers receiving an elementary education. Our findings are consistent with previous published studies; for instance, one study confirmed that ADHD was associated with lower educational achievement [37]. Moreover, the relationship between educational achievement and ADHD can be bidirectional; it is accepted that people with ADHD suffer from limitations in their academic performance. Furthermore, according to a study involving 548 people diagnosed with ADHD, higher education above high school was associated with lower odds for comorbid psychiatric disorders such as depression [38]. Conversely, the present study included refugees with a majority receiving a lower education level (elementary education); this could also explain the elevated percentage of ADHD screening (16%) compared to normal, undisplaced populations.

This pioneering study contributes significantly to the literature on adult ADHD symptoms in people with cardiovascular and related diseases living in crises. The sample type, sample size, and validated scales were the strengths of this study. However, this study had some limitations and challenges. For example, the cross-sectional design limits future follow-up, and the lack of specialized psychiatry prevents accurate diagnosis of ADHD in the study sample. The possibly exaggerated results for the consistency with ADHD symptoms are also a limitation, as in all the similar previous studies. However, the current study relied on a validated and reliable scale for ADHD, used a representative sample size, and carried out a solid statistical analysis, which reduced potential bias. Other limitations include the lack of control for other mental health issues, such as PTSD, anxiety, and depression. This can be included in future research.

Although the authors stress that it is not a proper diagnostic tool, this validated tool in Arabic-speaking populations provides insights for further personalized patient care services. In addition, the study did not examine important psychological aspects such as depression, anxiety, and stress that could contribute to ADHD. Furthermore, the current study did not explore ADHD symptoms in the context of a wider spectrum of cardiovascular disorders, such as metabolic syndrome. According to evidence, the examination of ADHD symptoms is better understood in the light of metabolic syndrome. This syndrome focuses on insulin resistance, dyslipidemia, and waist circumference, as well as diabetes [39]. Furthermore, data collection was a major challenge, as contacting the study participants was challenging, as a significant proportion of the phone numbers provided were disconnected, limiting data collection and sample size.

The findings of this study carry future implications on several dimensions regarding adult refugees with long-term diseases. The comprehensive patient care requires attentive addressing and regular follow-up for the potential clinical risk factors, such as disease severity, lack of medical supplies, and poor psychiatric care plans by specialists. This involves individualized service for ADHD and the other related disorders, such as PTSD, anxiety, and depression. The implementation of virtual psychiatric clinics can help overcome the infrastructure and logistical shortages and the social stigma towards mental health in this fragile population.

## 5. Conclusions

In conclusion, this study provided a preliminary screening of ADHD symptoms consistency and identified demographic and clinical associated factors faced by refugees affected by war-induced displacement. The high global prevalence of adult ADHD and the growing number of diagnoses highlight the importance of addressing healthcare issues in refugee settings. Although the study did not provide causal relationships with the outcome variable (ADHD screening), the associations presented provide important insights. These factors are crucial for gaining a deeper understanding of and effectively addressing the healthcare needs of refugee populations, who are simultaneously dealing with ADHD and cardiovascular conditions. These results highlight the necessity for healthcare interventions to mitigate the negative impacts of ADHD and long-term health conditions among refugees experiencing displacement due to crises.

## Figures and Tables

**Table 1:** Associations between each variable and the outcome variable (consistency with ADHD symptoms) for a cohort of refugees diagnosed with hypertension and diabetes (*n* = 345).

Variable	Category	Less Consistent with ADHD Symptoms *n* = 187 (54.2%)	Highly Consistent with ADHD Symptoms *n* = 158 (45.8%)	Chi-Square	*p*-Value
Sex	Male	93 (56%)	73 (44%)	0.42	0.52
	Female	94 (52.5%)	85 (47.5%)		
Age	Below 50 years	96 (60%)	64 (40%)	4.04	0.05
	>50 years	91 (49.2%)	94 (50.8%)		
Education	Elementary	114 (49.4%)	117 (50.6%)	5.98	0.01 *
	High school	71 (63.4%)	41 (36.6%)		
Residence	Amman	42 (56.8%)	32 (43.2%)	0.24	0.69
	Outside Amman	145 (53.5%)	126 (46.5%)		
Metformin	No	131 (62.1%)	80 (37.9%)	13.59	<0.001 *
	Yes	56 (41.8%)	78 (58.2%)		
Sulfonylurea	No	142 (54.8%)	117 (45.2%)	0.16	0.71
	Yes	45 (52.3%)	41 (47.7%)		
Insulin	No	177 (56.4%)	137 (43.6%)	6.60	0.01 *
	Yes	10 (32.3%)	21 (67.7%)		
Beta-blockers	No	104 (53.6%)	90 (46.4%)	0.06	0.82
	Yes	83 (55.0%)	68 (45.0%)		
ACEIs/ARBs	No	144 (56.9%)	109 (43.1%)	2.81	0.11
	Yes	43 (46.7%)	49 (53.3%)		
Diuretics	No	157 (55.5%)	126 (44.5%)	1.03	0.32
	Yes	30 (48.4%)	32 (51.6%)		
CCB	No	163 (55.8%)	129 (44.2%)	2.00	0.17
	Yes	24 (45.3%)	29 (54.7%)		

* *p* < 0.05.

**Table 2:** The final multivariable binary logistic regression analysis model for “consistency with ADHD symptoms” as the dependent variable, showing the significantly associated factors.

Independent Variable	B	Wald	*p*-Value	OR	Lower Estimate	Higher Estimate
High school education	−0.533	4.854	0.028	0.58	0.36	0.94
Metformin	0.735	10.196	0.001	2.08	1.32	3.27
Insulin	0.811	3.875	0.049	2.25	1.00	5.04
Constant	−0.349	4.464	0.035	0.70		

## Data Availability

Data associated with this research are available upon reasonable request from the authors.
